# Author Correction: 30×30 biodiversity gains rely on national coordination

**DOI:** 10.1038/s41467-023-43338-4

**Published:** 2023-11-27

**Authors:** Isaac Eckert, Andrea Brown, Dominique Caron, Federico Riva, Laura J. Pollock

**Affiliations:** 1 Dept. of Biology, McGill University, H3A 1B1 Montreal, QC Canada; 2 Quebec Center for Biodiversity Science, Montreal, QC Canada; 3 Institute for Environmental Studies, Vrije Universiteit Amsterdam, Amsterdam, The Netherlands

**Keywords:** Conservation biology, Biodiversity

Correction to: *Nature Communications* 10.1038/s41467-023-42737-x, published online 6 November 2023

The original version of this Article contained errors in panel b of Figure 2. Some of the orange bars representing “Protection gained (from current levels of protection)” and the superimposed text indicating the corresponding taxon were absent. This has been corrected in both the PDF and HTML versions of the Article.

