# Supplementary material for: 30×30 biodiversity gains rely on national coordination
Source: Nat Commun. 2023 Nov 6;14:7113. doi: 10.1038/s41467-023-42737-x (PMC10628259; doi:10.1038/s41467-023-42737-x)
Supplement: Supplementary file 1 — Supplementary Information [file 41467_2023_42737_MOESM1_ESM.pdf]

SUPPLEMENTARY INFORMATION:

Supplementary Table 1 — Model validation AUC and PRC scores for vertebrates, plants, and butterflies.

Kingdom	Region	Mean AUC	Sd AUC	Mean PRC	Sd PRC
Vertebrates	Canada	0.889	0.161	0.882	0.175
Vertebrates	North	0.893	0.124	0.874	0.141
Vertebrates	South	0.843	0.158	0.821	0.176
Plants	Canada	0.971	0.037	0.951	0.061
Plants	North	0.956	0.041	0.918	0.076
Plants	South	0.937	0.059	0.882	0.105
Butterflies	Canada	0.943	0.071	0.915	0.101
Butterflies	North	0.914	0.069	0.843	0.127
Butterflies	South	0.901	0.079	0.814	0.164

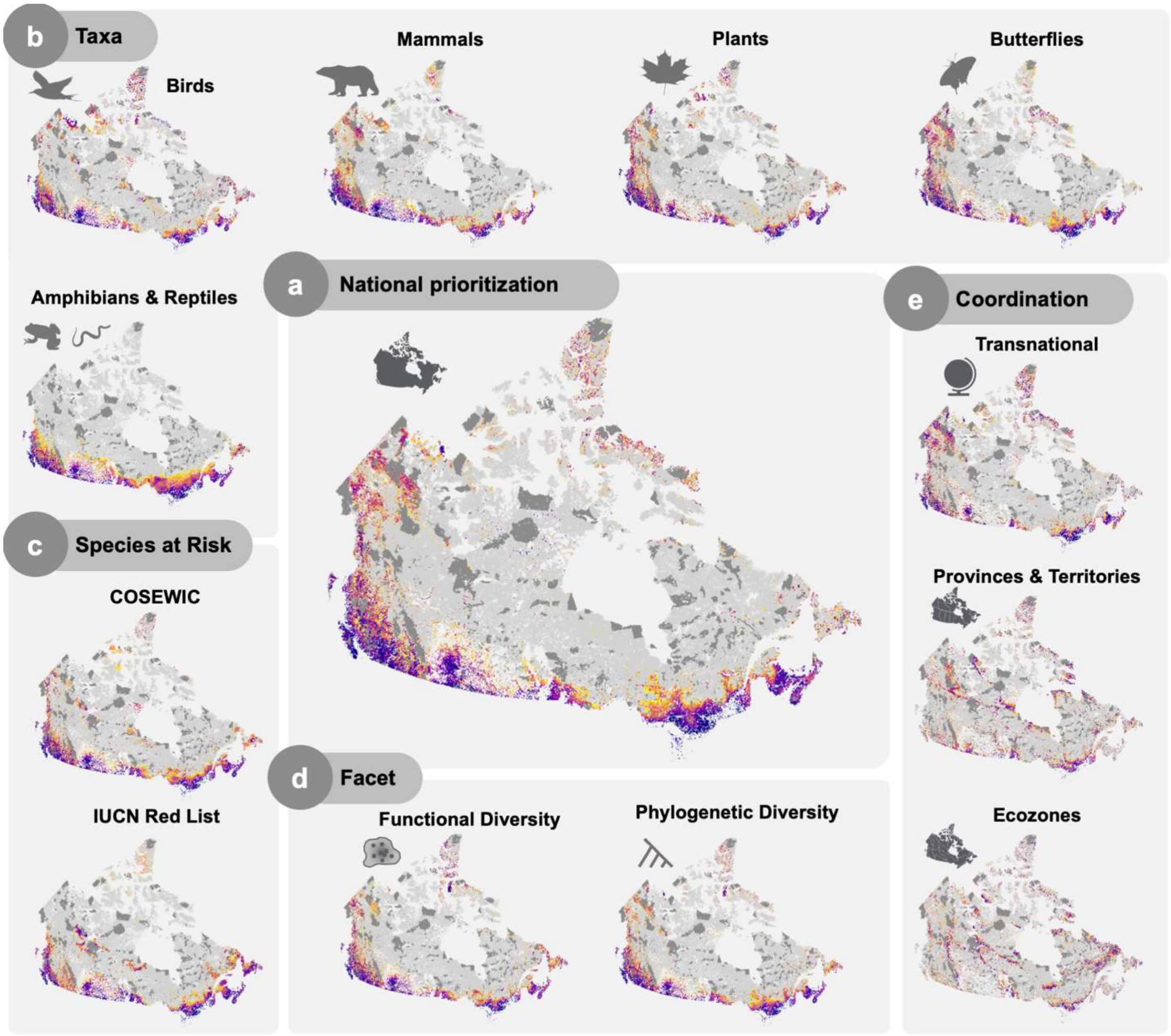

Supplementary Figure 1 — 30x30 prioritizations for all conservation priorities. The national prioritization (a) represents the optimum scenario for Canada, where all species, weighed equally across kingdoms, are prioritized at the national level, maximizing coverage across taxa. Taxa (b) prioritization scenarios include specific clades, Species at-Risk (c) prioritization scenarios include only species listed as at-risk by COSEWIC or IUCN Red List, and Facet (d) prioritization scenarios prioritize functional and phylogenetic diversity. Coordination (e) prioritization scenarios prioritize land differently across spatial scales, either prioritizing transnational diversity using species-specific weights based on endemism or Provincial &

Territorial and Ecozone diversity by performing separate prioritizations for each region, thereby achieving spatial representation. Land in the top 30% of each prioritization scenario is highlighted in color with blue cells representing the most important land, followed by red, and then yellow. Existing protected areas are colored in dark grey, and light grey land represents the 70% of Canada that falls outside 30x30 spatial priorities.

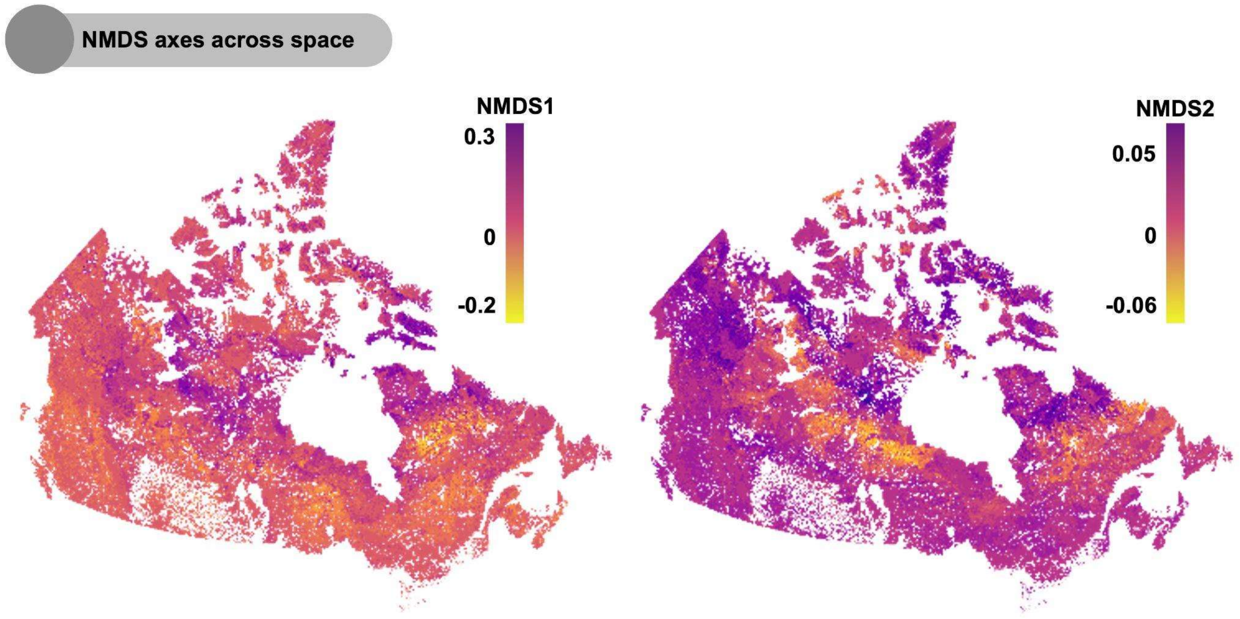

Supplementary Figure 2 — NMDS axes 1 and 2 mapped across space. These axes correspond to those in Fig. 5 and represent the top axes of variation in cell rank across all conservation prioritizations.
