# Supplementary material for: 30×30 biodiversity gains rely on national coordination
Source: Nat Commun. 2023 Nov 6;14:7113. doi: 10.1038/s41467-023-42737-x (PMC10628259; doi:10.1038/s41467-023-42737-x)
Supplement: Supplementary file 3 — Description of Additional Supplementary Files [file 41467_2023_42737_MOESM3_ESM.pdf]

Description of Additional Supplementary Files

File Name: Supplementary Data 1
Description: Different conservation scenarios protect different amounts of biodiversity. Table includes all conservation priorities, and reports the conservation gains possible under 30x30 as the percentage of that measure of biodiversity that would be adequately protected. Trade-offs were calculated as the difference between the potential for each conservation priority and the maximum potential for the national prioritization. A negative trade-off means that that conservation priority limits our ability to protect biodiversity compared to the national prioritization, while a positive trade-off indicates that that priority may protect biodiversity either as well, or better than the national prioritization. Positive trade-offs are the result of Zonation's marginal loss rule (CAZ2) that balances spatial priorities across species according to rarity and endemism. So, while we might be able to adequately protect more species by prioritizing amphibian & reptiles in spatial planning, the conservation gains would not be balanced across all taxa (for instance more northern species would be very poorly protected). Weighted endemism and SPI trade-offs were highly correlated (Pearson's $R=0.88$, $p<0.001$).
