# Supplementary material for: 30×30 biodiversity gains rely on national coordination
Source: Nat Commun. 2023 Nov 6;14:7113. doi: 10.1038/s41467-023-42737-x (PMC10628259; doi:10.1038/s41467-023-42737-x)
Supplement: Supplementary file 5 — Reporting Summary [file 41467_2023_42737_MOESM5_ESM.pdf]

nature portfolio

Corresponding author(s): Isaac Eckert

Last updated by author(s): Oct 11, 2023

Reporting Summary

Nature Portfolio wishes to improve the reproducibility of the work that we publish. This form provides structure for consistency and transparency in reporting. For further information on Nature Portfolio policies, see our Editorial Policies and the Editorial Policy Checklist.

Statistics

For all statistical analyses, confirm that the following items are present in the figure legend, table legend, main text, or Methods section.

n/a	Confirmed	
☐	☒	The exact sample size (*n*) for each experimental group/condition, given as a discrete number and unit of measurement
☒	☐	A statement on whether measurements were taken from distinct samples or whether the same sample was measured repeatedly
☐	☒	The statistical test(s) used AND whether they are one- or two-sided<br>*Only common tests should be described solely by name; describe more complex techniques in the Methods section.*
☒	☐	A description of all covariates tested
☒	☐	A description of any assumptions or corrections, such as tests of normality and adjustment for multiple comparisons
☐	☒	A full description of the statistical parameters including central tendency (e.g. means) or other basic estimates (e.g. regression coefficient) AND variation (e.g. standard deviation) or associated estimates of uncertainty (e.g. confidence intervals)
☐	☒	For null hypothesis testing, the test statistic (e.g. *F*, *t*, *r*) with confidence intervals, effect sizes, degrees of freedom and *P* value noted<br>*Give P values as exact values whenever suitable.*
☒	☐	For Bayesian analysis, information on the choice of priors and Markov chain Monte Carlo settings
☒	☐	For hierarchical and complex designs, identification of the appropriate level for tests and full reporting of outcomes
☐	☒	Estimates of effect sizes (e.g. Cohen's *d*, Pearson's *r*), indicating how they were calculated

Our web collection on statistics for biologists contains articles on many of the points above.

Software and code

Policy information about availability of computer code

Data collection	*Provide a description of all commercial, open source and custom code used to collect the data in this study, specifying the version used OR state that no software was used.*
Data analysis	We exclusively used R, R Studio, and Zonation 5. R and R Studio are free open source platforms and Zonation 5 is a free conservation planning software to run spatial prioritizations.

For manuscripts utilizing custom algorithms or software that are central to the research but not yet described in published literature, software must be made available to editors and reviewers. We strongly encourage code deposition in a community repository (e.g. GitHub). See the Nature Portfolio guidelines for submitting code & software for further information.

Data

Policy information about availability of data

All manuscripts must include a data availability statement. This statement should provide the following information, where applicable:

- Accession codes, unique identifiers, or web links for publicly available datasets
- A description of any restrictions on data availability
- For clinical datasets or third party data, please ensure that the statement adheres to our policy

We used a variety of R packages to write custom R code to access, collect, and clean data. We relied on large and well cited databases for species functional traits and phylogenies (all listed in the methods and publicly available). We also used a variety of spatial data layers all available Online and listed in the methods. All non-

publicly available data used in the analysis (which amounts to a few hand drawn spatial polygons used to filter geographic coordinates) are available in FigShare. All other data used is publicly available. In FigShare we also include our calculated functional, phylogenetic, and transnational weights, scenario rank maps in the following repository: https://figshare.com/s/0551e56687ba119c7bb8.

We used the following databases in this study:
- Global Biodiversity Information Facility (GBIF) observation data (https://www.gbif.org/)
- EButterfly observation data (https://www.e-butterfly.org/)
- IUCN Red List Range Polygons (https://www.iucnredlist.org/)
- Birds of the World Range Polygons (https://birdsoftheworld.org/bow/home)
- VASCAN list of vascular plants of Canada (https://data.canadensys.net/vascan/search)
- AdaptWest climate data (https://adaptwest.databasin.org/)
- MODIS land cover (https://modis.gsfc.nasa.gov/data/dataprod/mod12.php)
- Unified North American Soil Map (https://daac.ornl.gov/NACP/guides/NACP_MsTMIP_Unified_NA_SoilMap.html#:~:text=The%20Unified%20North%20American%20Soil,21).)
- Global Human Footprint (https://sedac.ciesin.columbia.edu/data/set/wildareas-v2-human-footprint-geographic)
- Canadian Human Footprint (https://www.facetsjournal.com/doi/10.1139/facets-2021-0063)
- Aboriginal lands of canada legislative boundaries (https://open.canada.ca/data/en/dataset/522b07b9-78e2-4819-b736-ad9208eb1067)
- Canada's ecological framework (https://open.canada.ca/data/en/dataset/7ad7ea01-eb23-4824-bccc-66adb7c5bdf8)
- Provinces & Territories of Canada (https://open.canada.ca/data/en/dataset/a883eb14-0c0e-45c4-b8c4-b54c4a819edb)
- Canadian Protected and Conserved Areas Database (https://www.canada.ca/en/environment-climate-change/services/national-wildlife-areas/protected-conserved-areas-database.html)
- COSEWIC status reports (https://www.cosewic.ca/index.php/en-ca/)
- Vertebrate Functional Data from AMNIOTE (https://esajournals.onlinelibrary.wiley.com/doi/10.1890/15-0846R.1), Amphibio (https://www.nature.com/articles/sdata2017123), panTHERIA (https://esajournals.onlinelibrary.wiley.com/doi/10.1890/08-1494.1)
- Plant functional data from TRY (https://try-db.org/TryWeb/Home.php)
- Butterfly functional data from (https://link.springer.com/article/10.1007/s10531-011-0088-y) and lepTraits (https://www.nature.com/articles/s41597-022-01473-5)
- phylogenetic data from VertLife (https://vertlife.org/), V.phylomaker, and (https://link.springer.com/article/10.1007/s10531-011-0088-y)

Research involving human participants, their data, or biological material

Policy information about studies with human participants or human data. See also policy information about sex, gender (identity/presentation), and sexual orientation and race, ethnicity and racism.

Reporting on sex and gender	NA
Reporting on race, ethnicity, or other socially relevant groupings	NA
Population characteristics	NA
Recruitment	NA
Ethics oversight	NA

Note that full information on the approval of the study protocol must also be provided in the manuscript.

Field-specific reporting

Please select the one below that is the best fit for your research. If you are not sure, read the appropriate sections before making your selection.

☐ Life sciences ☐ Behavioural & social sciences ☒ Ecological, evolutionary & environmental sciences

For a reference copy of the document with all sections, see nature.com/documents/nr-reporting-summary-flat.pdf

Ecological, evolutionary & environmental sciences study design

All studies must disclose on these points even when the disclosure is negative.

Study description	This study asks how prioritizing different elements of biodiversity or coordinating protection at different spatial scales impacts our ability to capture biodiversity in protected areas in the context of 30x30. To explore this question we focus on Canada as a model nation and model the distribution of all Canadian terrestrial vertebrates, plants, and butterflies using a robust species distribution modeling framework. We incorporate climate change into spatial planning by using foretasted future species distributions. We find that existing protected areas do not efficiently capture biodiversity but that meeting our 30x30 goals could protect over 65% of all Canadian biodiversity. How we coordinate protection across spatial scales has the largest impact on our ability to capture biodiversity in spatial planning. Notably, regional approaches that involve the spatial representation of protected areas protected much less

	biodiversity compared to nationally coordinated approaches.
Research sample	We used observations of all Canadian vertebrates, plants, and butterflies hosted on GBIF
Sampling strategy	All observations between specific years (1970-2021) were included in the analysis.
Data collection	Most data was publicly available and was downloaded using a computer. For some vertebrate species who lacked range polygons we hand drew polygons using Google Earth Pro, converting the JSON to a polygon in R.
Timing and spatial scale	Data was accessed over the course of a year depending on the data type (all species observations were downloaded within a single week period, functional and phylogeny data was accessed over the span of a few months). The spatial scale was for all of North America, but focusing on Canada for the study. All GBIF data was downloaded in May of 2021 to begin the modeling procedure. TRY plant functional data was downloaded in Oct of 2021. Data was only downloaded once.
Data exclusions	Species without enough data to model (detailed in the methods) were excluded from the analysis.
Reproducibility	We only used publicly available data with the exception of a few layers generated to filter coordinates which are available on FigShare. All code to reproduce the study will be published in FigShare upon acceptance. We cite all R packages and data sources in the methods to enable reproducibility.
Randomization	This is not relevant to our study since it is not experimental.
Blinding	Not relevant.

Did the study involve field work? ☐ Yes ☒ No

Reporting for specific materials, systems and methods

We require information from authors about some types of materials, experimental systems and methods used in many studies. Here, indicate whether each material, system or method listed is relevant to your study. If you are not sure if a list item applies to your research, read the appropriate section before selecting a response.

Materials & experimental systems

n/a	Involved in the study	
☒	☐	Antibodies
☒	☐	Eukaryotic cell lines
☒	☐	Palaeontology and archaeology
☒	☐	Animals and other organisms
☒	☐	Clinical data
☒	☐	Dual use research of concern
☒	☐	Plants

Methods

n/a	Involved in the study	
☒	☐	ChIP-seq
☒	☐	Flow cytometry
☒	☐	MRI-based neuroimaging
